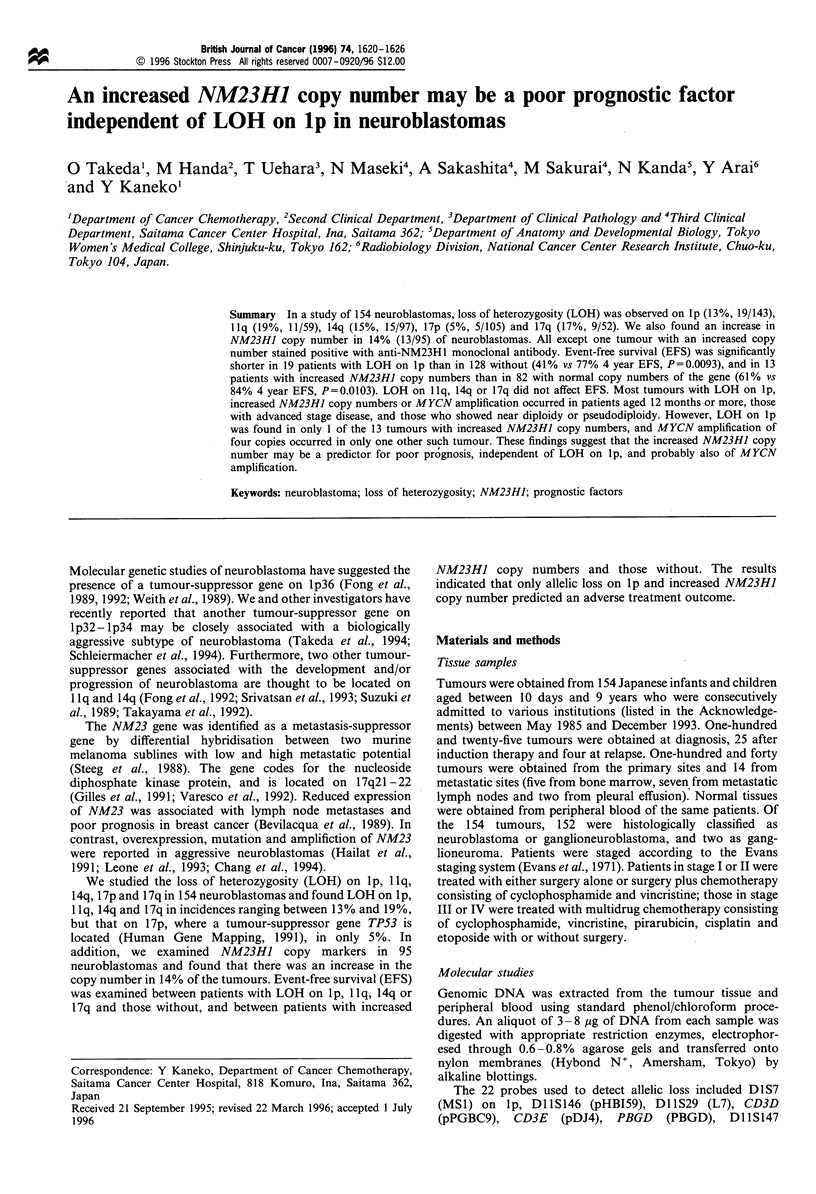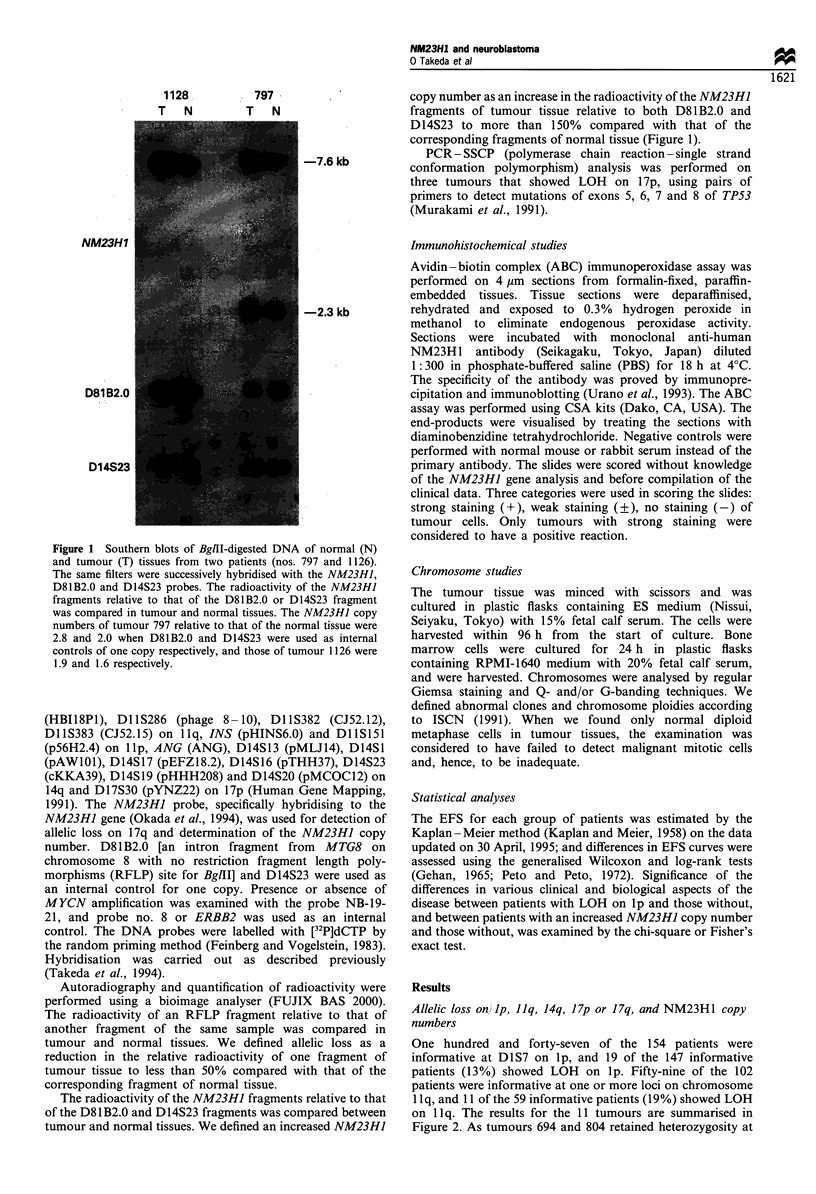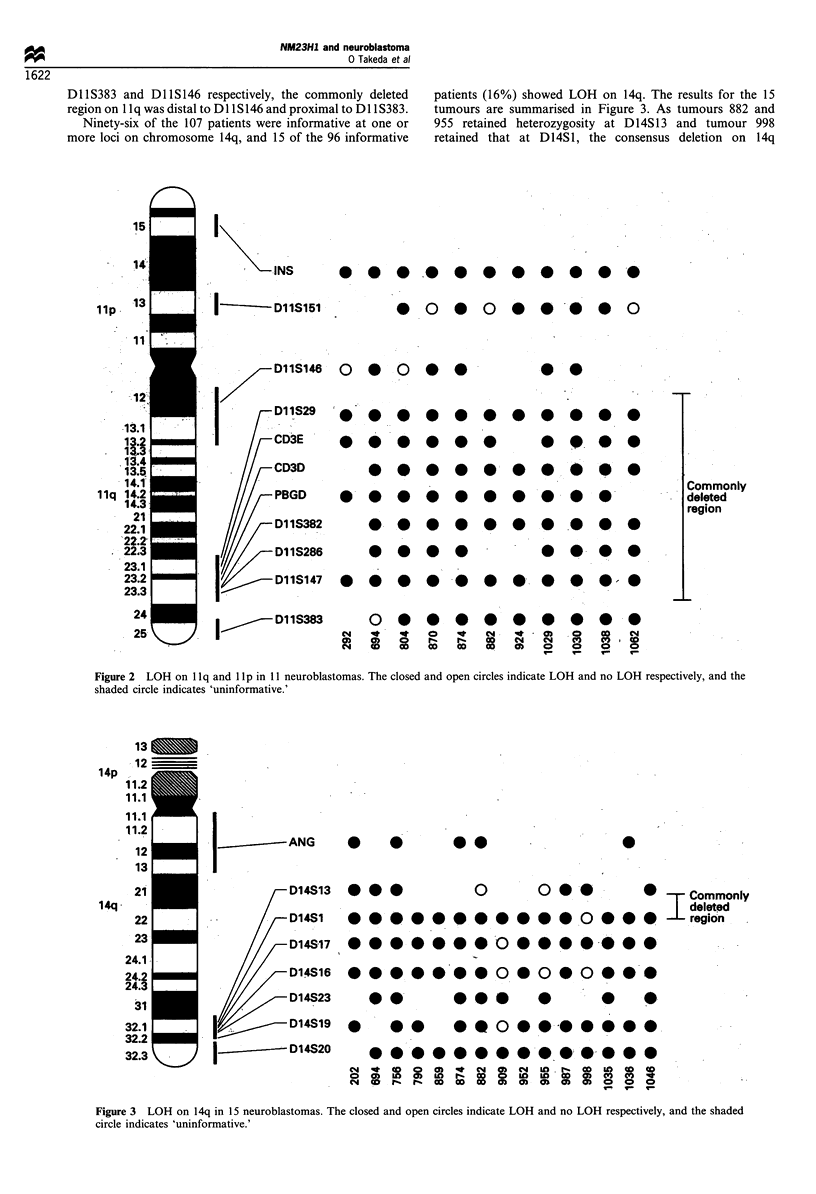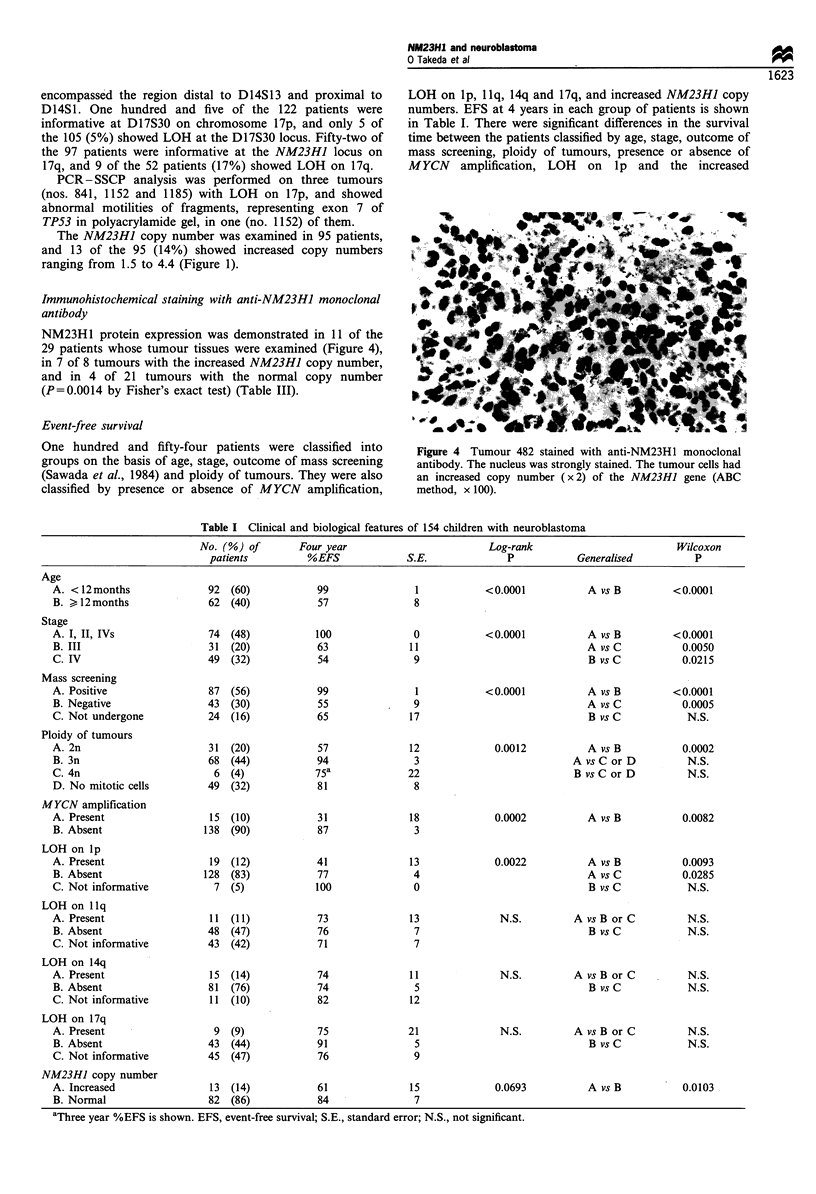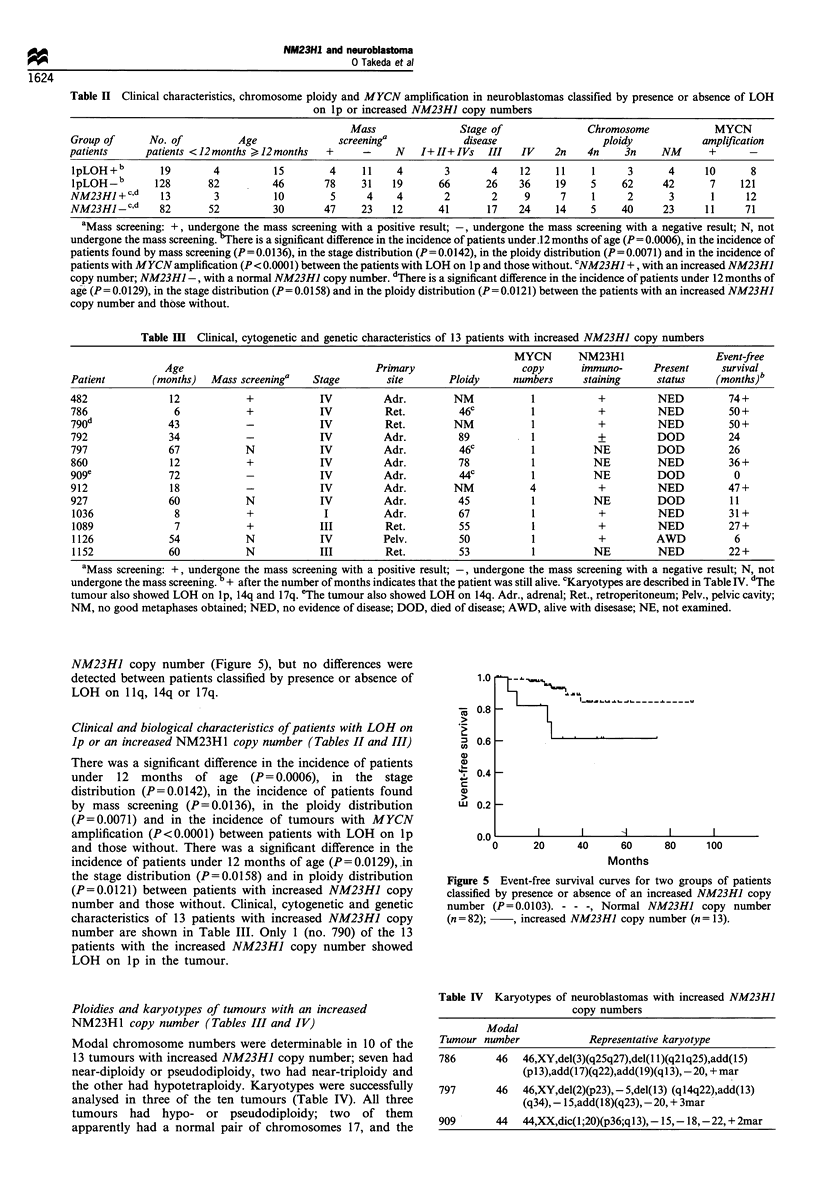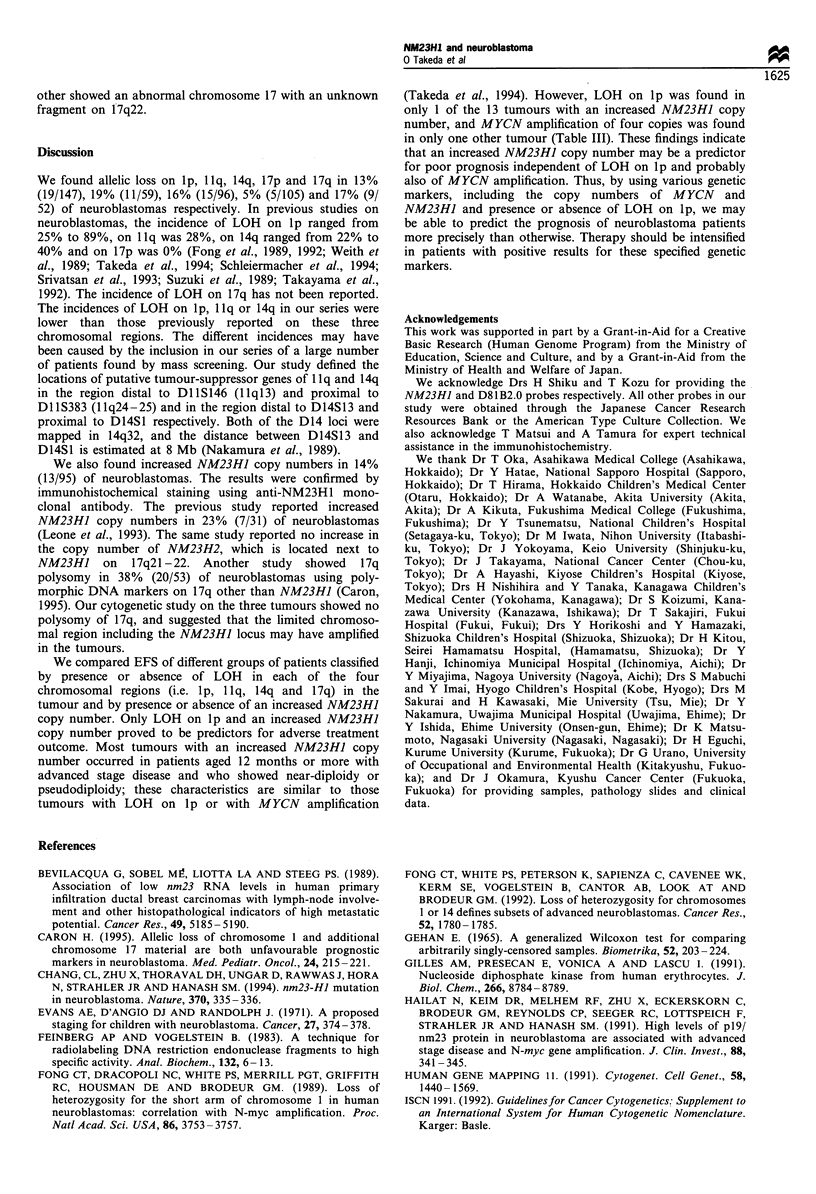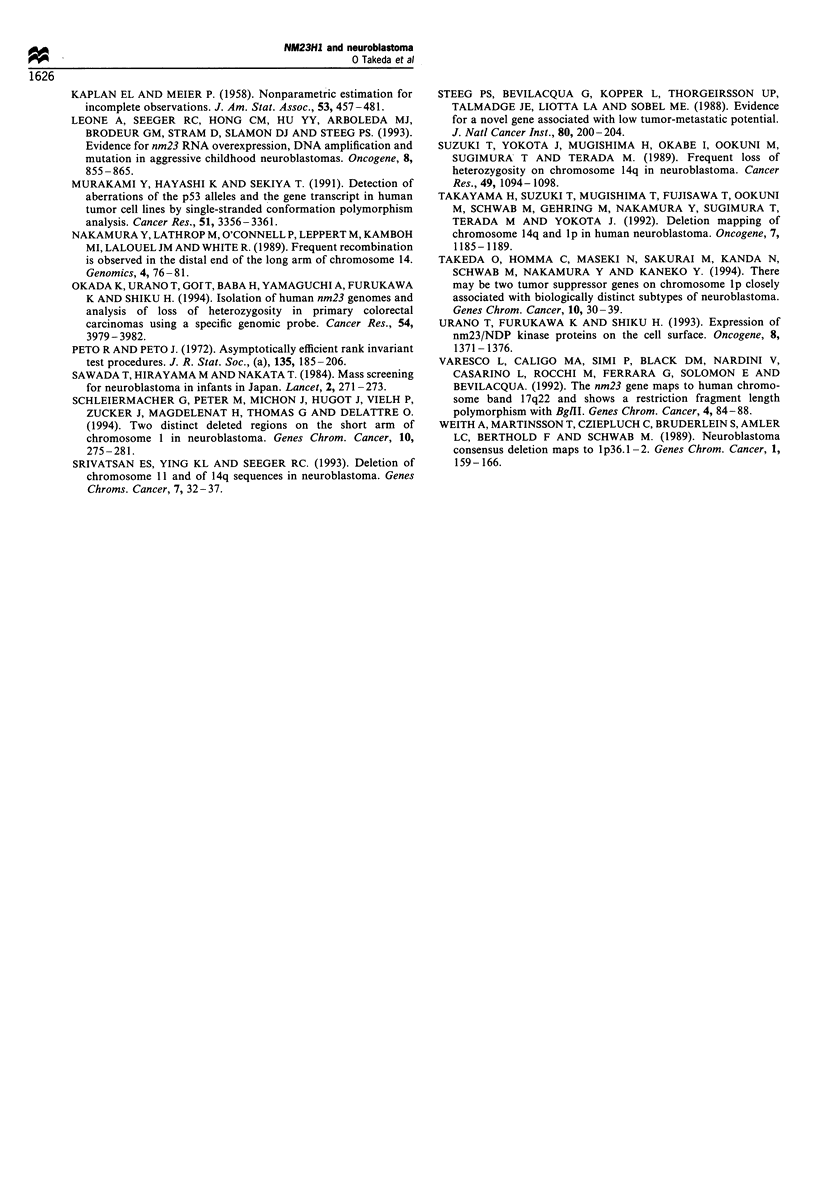# An increased NM23H1 copy number may be a poor prognostic factor independent of LOH on 1p in neuroblastomas.

**DOI:** 10.1038/bjc.1996.598

**Published:** 1996-11

**Authors:** O. Takeda, M. Handa, T. Uehara, N. Maseki, A. Sakashita, M. Sakurai, N. Kanda, Y. Arai, Y. Kaneko

**Affiliations:** Department of Cancer Chemotherapy, Saitama Cancer Center Hospital, Ina, Japan.

## Abstract

**Images:**


British Journal of Cancer (1996) 74, 1620-1626
© 1996 Stockton Press All rights reserved 0007-0920/96 $12.00

### An increased *NM23H1* copy number may be a poor prognostic factor independent of LOH on 1p in neuroblastomas

O Takeda[1], M Handa[2], T Uehara[3], N Maseki[4], A Sakashita[4], M Sakurai[4], N Kanda[5], Y Arai[6] and Y Kaneko[1]

[1]*Department of Cancer Chemotherapy,* [2]*Second Clinical Department,* [3]*Department of Clinical Pathology and* [4]*Third Clinical Department, Saitama Cancer Center Hospital, Ina, Saitama 362;* [5]*Department of Anatomy and Developmental Biology, Tokyo Women's Medical College, Shinjuku-ku, Tokyo 162;* [6]*Radiobiology Division, National Cancer Center Research Institute, Chuo-ku, Tokyo 104, Japan.*

**Summary** In a study of 154 neuroblastomas, loss of heterozygosity (LOH) was observed on 1p (13%, 19/143), 11q (19%, 11/59), 14q (15%, 15/97), 17p (5%, 5/105) and 17q (17%, 9/52). We also found an increase in *NM23H1* copy number in 14% (13/95) of neuroblastomas. All except one tumour with an increased copy number stained positive with anti-NM23H1 monoclonal antibody. Event-free survival (EFS) was significantly shorter in 19 patients with LOH on 1p than in 128 without (41% *vs* 77% 4 year EFS, $P=0.0093$), and in 13 patients with increased *NM23H1* copy numbers than in 82 with normal copy numbers of the gene (61% *vs* 84% 4 year EFS, $P=0.0103$). LOH on 11q, 14q or 17q did not affect EFS. Most tumours with LOH on 1p, increased *NM23H1* copy numbers or *MYCN* amplification occurred in patients aged 12 months or more, those with advanced stage disease, and those who showed near diploidy or pseudodiploidy. However, LOH on 1p was found in only 1 of the 13 tumours with increased *NM23H1* copy numbers, and *MYCN* amplification of four copies occurred in only one other such tumour. These findings suggest that the increased *NM23H1* copy number may be a predictor for poor prognosis, independent of LOH on 1p, and probably also of *MYCN* amplification.

**Keywords:** neuroblastoma; loss of heterozygosity; *NM23H1*; prognostic factors

Molecular genetic studies of neuroblastoma have suggested the presence of a tumour-suppressor gene on 1p36 (Fong *et al.*, 1989, 1992; Weith *et al.*, 1989). We and other investigators have recently reported that another tumour-suppressor gene on 1p32–1p34 may be closely associated with a biologically aggressive subtype of neuroblastoma (Takeda *et al.*, 1994; Schleiermacher *et al.*, 1994). Furthermore, two other tumour-suppressor genes associated with the development and/or progression of neuroblastoma are thought to be located on 11q and 14q (Fong *et al.*, 1992; Srivatsan *et al.*, 1993; Suzuki *et al.*, 1989; Takayama *et al.*, 1992).

The *NM23* gene was identified as a metastasis-suppressor gene by differential hybridisation between two murine melanoma sublines with low and high metastatic potential (Steeg *et al.*, 1988). The gene codes for the nucleoside diphosphate kinase protein, and is located on 17q21–22 (Gilles *et al.*, 1991; Varesco *et al.*, 1992). Reduced expression of *NM23* was associated with lymph node metastases and poor prognosis in breast cancer (Bevilacqua *et al.*, 1989). In contrast, overexpression, mutation and amplifiction of *NM23* were reported in aggressive neuroblastomas (Hailat *et al.*, 1991; Leone *et al.*, 1993; Chang *et al.*, 1994).

We studied the loss of heterozygosity (LOH) on 1p, 11q, 14q, 17p and 17q in 154 neuroblastomas and found LOH on 1p, 11q, 14q and 17q in incidences ranging between 13% and 19%, but that on 17p, where a tumour-suppressor gene *TP53* is located (Human Gene Mapping, 1991), in only 5%. In addition, we examined *NM23H1* copy markers in 95 neuroblastomas and found that there was an increase in the copy number in 14% of the tumours. Event-free survival (EFS) was examined between patients with LOH on 1p, 11q, 14q or 17q and those without, and between patients with increased *NM23H1* copy numbers and those without. The results indicated that only allelic loss on 1p and increased *NM23H1* copy number predicted an adverse treatment outcome.

Correspondence: Y Kaneko, Department of Cancer Chemotherapy, Saitama Cancer Center Hospital, 818 Komuro, Ina, Saitama 362, Japan
Received 21 September 1995; revised 22 March 1996; accepted 1 July 1996

### Materials and methods

### *Tissue samples*

Tumours were obtained from 154 Japanese infants and children aged between 10 days and 9 years who were consecutively admitted to various institutions (listed in the Acknowledgements) between May 1985 and December 1993. One-hundred and twenty-five tumours were obtained at diagnosis, 25 after induction therapy and four at relapse. One-hundred and forty tumours were obtained from the primary sites and 14 from metastatic sites (five from bone marrow, seven from metastatic lymph nodes and two from pleural effusion). Normal tissues were obtained from peripheral blood of the same patients. Of the 154 tumours, 152 were histologically classified as neuroblastoma or ganglioneuroblastoma, and two as ganglioneuroma. Patients were staged according to the Evans staging system (Evans *et al.*, 1971). Patients in stage I or II were treated with either surgery alone or surgery plus chemotherapy consisting of cyclophosphamide and vincristine; those in stage III or IV were treated with multidrug chemotherapy consisting of cyclophosphamide, vincristine, pirarubicin, cisplatin and etoposide with or without surgery.

### *Molecular studies*

Genomic DNA was extracted from the tumour tissue and peripheral blood using standard phenol/chloroform procedures. An aliquot of 3–8 μg of DNA from each sample was digested with appropriate restriction enzymes, electrophoresed through 0.6–0.8% agarose gels and transferred onto nylon membranes (Hybond $N^+$, Amersham, Tokyo) by alkaline blottings.

The 22 probes used to detect allelic loss included D1S7 (MS1) on 1p, D11S146 (pHBI59), D11S29 (L7), *CD3D* (pPGBC9), *CD3E* (pDJ4), *PBGD* (PBGD), D11S147

**Figure 1** Southern blots of *Bgl*II-digested DNA of normal (N) and tumour (T) tissues from two patients (nos. 797 and 1126). The same filters were successively hybridised with the *NM23H1*, D81B2.0 and D14S23 probes. The radioactivity of the *NM23H1* fragments relative to that of the D81B2.0 or D14S23 fragment was compared in tumour and normal tissues. The *NM23H1* copy numbers of tumour 797 relative to that of the normal tissue were 2.8 and 2.0 when D81B2.0 and D14S23 were used as internal controls of one copy respectively, and those of tumour 1126 were 1.9 and 1.6 respectively.

(HBI18P1), D11S286 (phage 8–10), D11S382 (CJ52.12), D11S383 (CJ52.15) on 11q, *INS* (pHINS6.0) and D11S151 (p56H2.4) on 11p, *ANG* (ANG), D14S13 (pMLJ14), D14S1 (pAW101), D14S17 (pEFZ18.2), D14S16 (pTHH37), D14S23 (cKKA39), D14S19 (pHHH208) and D14S20 (pMCOC12) on 14q and D17S30 (pYNZ22) on 17p (Human Gene Mapping, 1991). The *NM23H1* probe, specifically hybridising to the *NM23H1* gene (Okada *et al.*, 1994), was used for detection of allelic loss on 17q and determination of the *NM23H1* copy number. D81B2.0 [an intron fragment from *MTG8* on chromosome 8 with no restriction fragment length polymorphisms (RFLP) site for *Bgl*II] and D14S23 were used as an internal control for one copy. Presence or absence of *MYCN* amplification was examined with the probe NB-19-21, and probe no. 8 or *ERBB2* was used as an internal control. The DNA probes were labelled with [$^{32}$P]dCTP by the random priming method (Feinberg and Vogelstein, 1983). Hybridisation was carried out as described previously (Takeda *et al.*, 1994).

Autoradiography and quantification of radioactivity were performed using a bioimage analyser (FUJIX BAS 2000). The radioactivity of an RFLP fragment relative to that of another fragment of the same sample was compared in tumour and normal tissues. We defined allelic loss as a reduction in the relative radioactivity of one fragment of tumour tissue to less than 50% compared with that of the corresponding fragment of normal tissue.

The radioactivity of the *NM23H1* fragments relative to that of the D81B2.0 and D14S23 fragments was compared between tumour and normal tissues. We defined an increased *NM23H1* copy number as an increase in the radioactivity of the *NM23H1* fragments of tumour tissue relative to both D81B2.0 and D14S23 to more than 150% compared with that of the corresponding fragments of normal tissue (Figure 1).

PCR–SSCP (polymerase chain reaction–single strand conformation polymorphism) analysis was performed on three tumours that showed LOH on 17p, using pairs of primers to detect mutations of exons 5, 6, 7 and 8 of *TP53* (Murakami *et al.*, 1991).

### *Immunohistochemical studies*

Avidin–biotin complex (ABC) immunoperoxidase assay was performed on 4 μm sections from formalin-fixed, paraffin-embedded tissues. Tissue sections were deparaffinised, rehydrated and exposed to 0.3% hydrogen peroxide in methanol to eliminate endogenous peroxidase activity. Sections were incubated with monoclonal anti-human NM23H1 antibody (Seikagaku, Tokyo, Japan) diluted 1:300 in phosphate-buffered saline (PBS) for 18 h at 4°C. The specificity of the antibody was proved by immunoprecipitation and immunoblotting (Urano *et al.*, 1993). The ABC assay was performed using CSA kits (Dako, CA, USA). The end-products were visualised by treating the sections with diaminobenzidine tetrahydrochloride. Negative controls were performed with normal mouse or rabbit serum instead of the primary antibody. The slides were scored without knowledge of the *NM23H1* gene analysis and before compilation of the clinical data. Three categories were used in scoring the slides: strong staining (+), weak staining (±), no staining (−) of tumour cells. Only tumours with strong staining were considered to have a positive reaction.

### *Chromosome studies*

The tumour tissue was minced with scissors and was cultured in plastic flasks containing ES medium (Nissui, Seiyaku, Tokyo) with 15% fetal calf serum. The cells were harvested within 96 h from the start of culture. Bone marrow cells were cultured for 24 h in plastic flasks containing RPMI-1640 medium with 20% fetal calf serum, and were harvested. Chromosomes were analysed by regular Giemsa staining and Q- and/or G-banding techniques. We defined abnormal clones and chromosome ploidies according to ISCN (1991). When we found only normal diploid metaphase cells in tumour tissues, the examination was considered to have failed to detect malignant mitotic cells and, hence, to be inadequate.

### *Statistical analyses*

The EFS for each group of patients was estimated by the Kaplan–Meier method (Kaplan and Meier, 1958) on the data updated on 30 April, 1995; and differences in EFS curves were assessed using the generalised Wilcoxon and log-rank tests (Gehan, 1965; Peto and Peto, 1972). Significance of the differences in various clinical and biological aspects of the disease between patients with LOH on 1p and those without, and between patients with an increased *NM23H1* copy number and those without, was examined by the chi-square or Fisher's exact test.

### Results

### *Allelic loss on 1p, 11q, 14q, 17p or 17q, and* NM23H1 *copy numbers*

One hundred and forty-seven of the 154 patients were informative at D1S7 on 1p, and 19 of the 147 informative patients (13%) showed LOH on 1p. Fifty-nine of the 102 patients were informative at one or more loci on chromosome 11q, and 11 of the 59 informative patients (19%) showed LOH on 11q. The results for the 11 tumours are summarised in Figure 2. As tumours 694 and 804 retained heterozygosity at

D11S383 and D11S146 respectively, the commonly deleted region on 11q was distal to D11S146 and proximal to D11S383.

Ninety-six of the 107 patients were informative at one or more loci on chromosome 14q, and 15 of the 96 informative patients (16%) showed LOH on 14q. The results for the 15 tumours are summarised in Figure 3. As tumours 882 and 955 retained heterozygosity at D14S13 and tumour 998 retained that at D14S1, the consensus deletion on 14q

**Figure 2** LOH on 11q and 11p in 11 neuroblastomas. The closed and open circles indicate LOH and no LOH respectively, and the shaded circle indicates 'uninformative.'

**Figure 3** LOH on 14q in 15 neuroblastomas. The closed and open circles indicate LOH and no LOH respectively, and the shaded circle indicates 'uninformative.'

encompassed the region distal to D14S13 and proximal to D14S1. One hundred and five of the 122 patients were informative at D17S30 on chromosome 17p, and only 5 of the 105 (5%) showed LOH at the D17S30 locus. Fifty-two of the 97 patients were informative at the *NM23H1* locus on 17q, and 9 of the 52 patients (17%) showed LOH on 17q.

PCR-SSCP analysis was performed on three tumours (nos. 841, 1152 and 1185) with LOH on 17p, and showed abnormal motilities of fragments, representing exon 7 of *TP53* in polyacrylamide gel, in one (no. 1152) of them.

The *NM23H1* copy number was examined in 95 patients, and 13 of the 95 (14%) showed increased copy numbers ranging from 1.5 to 4.4 (Figure 1).

*Immunohistochemical staining with anti-NM23H1 monoclonal antibody*

NM23H1 protein expression was demonstrated in 11 of the 29 patients whose tumour tissues were examined (Figure 4), in 7 of 8 tumours with the increased *NM23H1* copy number, and in 4 of 21 tumours with the normal copy number ($P = 0.0014$ by Fisher's exact test) (Table III).

*Event-free survival*

One hundred and fifty-four patients were classified into groups on the basis of age, stage, outcome of mass screening (Sawada *et al.*, 1984) and ploidy of tumours. They were also classified by presence or absence of *MYCN* amplification, LOH on 1p, 11q, 14q and 17q, and increased *NM23H1* copy numbers. EFS at 4 years in each group of patients is shown in Table I. There were significant differences in the survival time between the patients classified by age, stage, outcome of mass screening, ploidy of tumours, presence or absence of *MYCN* amplification, LOH on 1p and the increased

**Figure 4** Tumour 482 stained with anti-NM23H1 monoclonal antibody. The nucleus was strongly stained. The tumour cells had an increased copy number (×2) of the *NM23H1* gene (ABC method, ×100).

**Table I** Clinical and biological features of 154 children with neuroblastoma

| | *No. (%) of patients* | *Four year %EFS* | *S.E.* | *Log-rank* P | *Generalised* | *Wilcoxon* P |
|---|---|---|---|---|---|---|
| Age | | | | | | |
| A. <12 months | 92 (60) | 99 | 1 | <0.0001 | A *vs* B | <0.0001 |
| B. ≥12 months | 62 (40) | 57 | 8 | | | |
| Stage | | | | | | |
| A. I, II, IVs | 74 (48) | 100 | 0 | <0.0001 | A *vs* B | <0.0001 |
| B. III | 31 (20) | 63 | 11 | | A *vs* C | 0.0050 |
| C. IV | 49 (32) | 54 | 9 | | B *vs* C | 0.0215 |
| Mass screening | | | | | | |
| A. Positive | 87 (56) | 99 | 1 | <0.0001 | A *vs* B | <0.0001 |
| B. Negative | 43 (30) | 55 | 9 | | A *vs* C | 0.0005 |
| C. Not undergone | 24 (16) | 65 | 17 | | B *vs* C | N.S. |
| Ploidy of tumours | | | | | | |
| A. 2n | 31 (20) | 57 | 12 | 0.0012 | A *vs* B | 0.0002 |
| B. 3n | 68 (44) | 94 | 3 | | A *vs* C or D | N.S. |
| C. 4n | 6 (4) | 75[a] | 22 | | B *vs* C or D | N.S. |
| D. No mitotic cells | 49 (32) | 81 | 8 | | | |
| *MYCN* amplification | | | | | | |
| A. Present | 15 (10) | 31 | 18 | 0.0002 | A *vs* B | 0.0082 |
| B. Absent | 138 (90) | 87 | 3 | | | |
| LOH on 1p | | | | | | |
| A. Present | 19 (12) | 41 | 13 | 0.0022 | A *vs* B | 0.0093 |
| B. Absent | 128 (83) | 77 | 4 | | A *vs* C | 0.0285 |
| C. Not informative | 7 (5) | 100 | 0 | | B *vs* C | N.S. |
| LOH on 11q | | | | | | |
| A. Present | 11 (11) | 73 | 13 | N.S. | A *vs* B or C | N.S. |
| B. Absent | 48 (47) | 76 | 7 | | B *vs* C | N.S. |
| C. Not informative | 43 (42) | 71 | 7 | | | |
| LOH on 14q | | | | | | |
| A. Present | 15 (14) | 74 | 11 | N.S. | A *vs* B or C | N.S. |
| B. Absent | 81 (76) | 74 | 5 | | B *vs* C | N.S. |
| C. Not informative | 11 (10) | 82 | 12 | | | |
| LOH on 17q | | | | | | |
| A. Present | 9 (9) | 75 | 21 | N.S. | A *vs* B or C | N.S. |
| B. Absent | 43 (44) | 91 | 5 | | B *vs* C | N.S. |
| C. Not informative | 45 (47) | 76 | 9 | | | |
| *NM23H1* copy number | | | | | | |
| A. Increased | 13 (14) | 61 | 15 | 0.0693 | A *vs* B | 0.0103 |
| B. Normal | 82 (86) | 84 | 7 | | | |

[a]Three year %EFS is shown. EFS, event-free survival; S.E., standard error; N.S., not significant.

**Table II** Clinical characteristics, chromosome ploidy and *MYCN* amplification in neuroblastomas classified by presence or absence of LOH on 1p or increased *NM23H1* copy numbers

| *Group of patients* | *No. of patients* | *Age* <12 *months* | *Age* ≥12 *months* | *Mass screening*[a] + | − | *N* | *Stage of disease* *I+II+IVs* | *III* | *IV* | *Chromosome ploidy* *2n* | *4n* | *3n* | *NM* | MYCN *amplification* + | − |
|---|---|---|---|---|---|---|---|---|---|---|---|---|---|---|---|
| 1pLOH+[b] | 19 | 4 | 15 | 4 | 11 | 4 | 3 | 4 | 12 | 11 | 1 | 3 | 4 | 10 | 8 |
| 1pLOH−[b] | 128 | 82 | 46 | 78 | 31 | 19 | 66 | 26 | 36 | 19 | 5 | 62 | 42 | 7 | 121 |
| *NM23H1*+[c,d] | 13 | 3 | 10 | 5 | 4 | 4 | 2 | 2 | 9 | 7 | 1 | 2 | 3 | 1 | 12 |
| *NM23H1*−[c,d] | 82 | 52 | 30 | 47 | 23 | 12 | 41 | 17 | 24 | 14 | 5 | 40 | 23 | 11 | 71 |

[a]Mass screening: +, undergone the mass screening with a positive result; −, undergone the mass screening with a negative result; N, not undergone the mass screening. [b]There is a significant difference in the incidence of patients under 12 months of age ($P=0.0006$), in the incidence of patients found by mass screening ($P=0.0136$), in the stage distribution ($P=0.0142$), in the ploidy distribution ($P=0.0071$) and in the incidence of patients with *MYCN* amplification ($P<0.0001$) between the patients with LOH on 1p and those without. [c]*NM23H1*+, with an increased *NM23H1* copy number; *NM23H1*−, with a normal *NM23H1* copy number. [d]There is a significant difference in the incidence of patients under 12 months of age ($P=0.0129$), in the stage distribution ($P=0.0158$) and in the ploidy distribution ($P=0.0121$) between the patients with an increased *NM23H1* copy number and those without.

**Table III** Clinical, cytogenetic and genetic characteristics of 13 patients with increased *NM23H1* copy numbers

| *Patient* | *Age (months)* | *Mass screening*[a] | *Stage* | *Primary site* | *Ploidy* | MYCN *copy numbers* | NM23H1 *immuno-staining* | *Present status* | *Event-free survival (months)*[b] |
|---|---|---|---|---|---|---|---|---|---|
| 482 | 12 | + | IV | Adr. | NM | 1 | + | NED | 74+ |
| 786 | 6 | + | IV | Ret. | 46[c] | 1 | + | NED | 50+ |
| 790[d] | 43 | − | IV | Ret. | NM | 1 | + | NED | 50+ |
| 792 | 34 | − | IV | Adr. | 89 | 1 | ± | DOD | 24 |
| 797 | 67 | N | IV | Adr. | 46[c] | 1 | NE | DOD | 26 |
| 860 | 12 | + | IV | Adr. | 78 | 1 | NE | NED | 36+ |
| 909[e] | 72 | − | IV | Adr. | 44[c] | 1 | NE | DOD | 0 |
| 912 | 18 | − | IV | Adr. | NM | 4 | + | NED | 47+ |
| 927 | 60 | N | IV | Adr. | 45 | 1 | NE | DOD | 11 |
| 1036 | 8 | + | I | Adr. | 67 | 1 | + | NED | 31+ |
| 1089 | 7 | + | III | Ret. | 55 | 1 | + | NED | 27+ |
| 1126 | 54 | N | IV | Pelv. | 50 | 1 | + | AWD | 6 |
| 1152 | 60 | N | III | Ret. | 53 | 1 | NE | NED | 22+ |

[a]Mass screening: +, undergone the mass screening with a positive result; −, undergone the mass screening with a negative result; N, not undergone the mass screening. [b]+ after the number of months indicates that the patient was still alive. [c]Karyotypes are described in Table IV. [d]The tumour also showed LOH on 1p, 14q and 17q. [e]The tumour also showed LOH on 14q. Adr., adrenal; Ret., retroperitoneum; Pelv., pelvic cavity; NM, no good metaphases obtained; NED, no evidence of disease; DOD, died of disease; AWD, alive with disesase; NE, not examined.

*NM23H1* copy number (Figure 5), but no differences were detected between patients classified by presence or absence of LOH on 11q, 14q or 17q.

### *Clinical and biological characteristics of patients with LOH on 1p or an increased* NM23H1 *copy number (Tables II and III)*

There was a significant difference in the incidence of patients under 12 months of age ($P=0.0006$), in the stage distribution ($P=0.0142$), in the incidence of patients found by mass screening ($P=0.0136$), in the ploidy distribution ($P=0.0071$) and in the incidence of tumours with *MYCN* amplification ($P<0.0001$) between patients with LOH on 1p and those without. There was a significant difference in the incidence of patients under 12 months of age ($P=0.0129$), in the stage distribution ($P=0.0158$) and in ploidy distribution ($P=0.0121$) between patients with increased *NM23H1* copy number and those without. Clinical, cytogenetic and genetic characteristics of 13 patients with increased *NM23H1* copy number are shown in Table III. Only 1 (no. 790) of the 13 patients with the increased *NM23H1* copy number showed LOH on 1p in the tumour.

**Figure 5** Event-free survival curves for two groups of patients classified by presence or absence of an increased *NM23H1* copy number ($P=0.0103$). - - -, Normal *NM23H1* copy number ($n=82$); ——, increased *NM23H1* copy number ($n=13$).

### *Ploidies and karyotypes of tumours with an increased* NM23H1 *copy number (Tables III and IV)*

Modal chromosome numbers were determinable in 10 of the 13 tumours with increased *NM23H1* copy number; seven had near-diploidy or pseudodiploidy, two had near-triploidy and the other had hypotetraploidy. Karyotypes were successfully analysed in three of the ten tumours (Table IV). All three tumours had hypo- or pseudodiploidy; two of them apparently had a normal pair of chromosomes 17, and the

**Table IV** Karyotypes of neuroblastomas with increased *NM23H1* copy numbers

| *Tumour* | *Modal number* | *Representative karyotype* |
|---|---|---|
| 786 | 46 | 46,XY,del(3)(q25q27),del(11)(q21q25),add(15)(p13),add(17)(q22),add(19)(q13),−20,+mar |
| 797 | 46 | 46,XY,del(2)(p23),−5,del(13) (q14q22),add(13)(q34),−15,add(18)(q23),−20,+3mar |
| 909 | 44 | 44,XX,dic(1;20)(p36;q13),−15,−18,−22,+2mar |

other showed an abnormal chromosome 17 with an unknown fragment on 17q22.

### Discussion

We found allelic loss on 1p, 11q, 14q, 17p and 17q in 13% (19/147), 19% (11/59), 16% (15/96), 5% (5/105) and 17% (9/52) of neuroblastomas respectively. In previous studies on neuroblastomas, the incidence of LOH on 1p ranged from 25% to 89%, on 11q was 28%, on 14q ranged from 22% to 40% and on 17p was 0% (Fong *et al.*, 1989, 1992; Weith *et al.*, 1989; Takeda *et al.*, 1994; Schleiermacher *et al.*, 1994; Srivatsan *et al.*, 1993; Suzuki *et al.*, 1989; Takayama *et al.*, 1992). The incidence of LOH on 17q has not been reported. The incidences of LOH on 1p, 11q or 14q in our series were lower than those previously reported on these three chromosomal regions. The different incidences may have been caused by the inclusion in our series of a large number of patients found by mass screening. Our study defined the locations of putative tumour-suppressor genes of 11q and 14q in the region distal to D11S146 (11q13) and proximal to D11S383 (11q24–25) and in the region distal to D14S13 and proximal to D14S1 respectively. Both of the D14 loci were mapped in 14q32, and the distance between D14S13 and D14S1 is estimated at 8 Mb (Nakamura *et al.*, 1989).

We also found increased *NM23H1* copy numbers in 14% (13/95) of neuroblastomas. The results were confirmed by immunohistochemical staining using anti-NM23H1 monoclonal antibody. The previous study reported increased *NM23H1* copy numbers in 23% (7/31) of neuroblastomas (Leone *et al.*, 1993). The same study reported no increase in the copy number of *NM23H2*, which is located next to *NM23H1* on 17q21–22. Another study showed 17q polysomy in 38% (20/53) of neuroblastomas using polymorphic DNA markers on 17q other than *NM23H1* (Caron, 1995). Our cytogenetic study on the three tumours showed no polysomy of 17q, and suggested that the limited chromosomal region including the *NM23H1* locus may have amplified in the tumours.

We compared EFS of different groups of patients classified by presence or absence of LOH in each of the four chromosomal regions (i.e. 1p, 11q, 14q and 17q) in the tumour and by presence or absence of an increased *NM23H1* copy number. Only LOH on 1p and an increased *NM23H1* copy number proved to be predictors for adverse treatment outcome. Most tumours with an increased *NM23H1* copy number occurred in patients aged 12 months or more with advanced stage disease and who showed near-diploidy or pseudodiploidy; these characteristics are similar to those tumours with LOH on 1p or with *MYCN* amplification (Takeda *et al.*, 1994). However, LOH on 1p was found in only 1 of the 13 tumours with an increased *NM23H1* copy number, and *MYCN* amplification of four copies was found in only one other tumour (Table III). These findings indicate that an increased *NM23H1* copy number may be a predictor for poor prognosis independent of LOH on 1p and probably also of *MYCN* amplification. Thus, by using various genetic markers, including the copy numbers of *MYCN* and *NM23H1* and presence or absence of LOH on 1p, we may be able to predict the prognosis of neuroblastoma patients more precisely than otherwise. Therapy should be intensified in patients with positive results for these specified genetic markers.

**Acknowledgements**
This work was supported in part by a Grant-in-Aid for a Creative Basic Research (Human Genome Program) from the Ministry of Education, Science and Culture, and by a Grant-in-Aid from the Ministry of Health and Welfare of Japan.

We acknowledge Drs H Shiku and T Kozu for providing the *NM23H1* and D81B2.0 probes respectively. All other probes in our study were obtained through the Japanese Cancer Research Resources Bank or the American Type Culture Collection. We also acknowledge T Matsui and A Tamura for expert technical assistance in the immunohistochemistry.

We thank Dr T Oka, Asahikawa Medical College (Asahikawa, Hokkaido); Dr Y Hatae, National Sapporo Hospital (Sapporo, Hokkaido); Dr T Hirama, Hokkaido Children's Medical Center (Otaru, Hokkaido); Dr A Watanabe, Akita University (Akita, Akita); Dr A Kikuta, Fukushima Medical College (Fukushima, Fukushima); Dr Y Tsunematsu, National Children's Hospital (Setagaya-ku, Tokyo); Dr M Iwata, Nihon University (Itabashi-ku, Tokyo); Dr J Yokoyama, Keio University (Shinjuku-ku, Tokyo); Dr J Takayama, National Cancer Center (Chou-ku, Tokyo); Dr A Hayashi, Kiyose Children's Hospital (Kiyose, Tokyo); Drs H Nishihira and Y Tanaka, Kanagawa Children's Medical Center (Yokohama, Kanagawa); Dr S Koizumi, Kanazawa University (Kanazawa, Ishikawa); Dr T Sakajiri, Fukui Hospital (Fukui, Fukui); Drs Y Horikoshi and Y Hamazaki, Shizuoka Children's Hospital (Shizuoka, Shizuoka); Dr H Kitou, Seirei Hamamatsu Hospital, (Hamamatsu, Shizuoka); Dr Y Hanji, Ichinomiya Municipal Hospital (Ichinomiya, Aichi); Dr Y Miyajima, Nagoya University (Nagoya, Aichi); Drs S Mabuchi and Y Imai, Hyogo Children's Hospital (Kobe, Hyogo); Drs M Sakurai and H Kawasaki, Mie University (Tsu, Mie); Dr Y Nakamura, Uwajima Municipal Hospital (Uwajima, Ehime); Dr Y Ishida, Ehime University (Onsen-gun, Ehime); Dr K Matsumoto, Nagasaki University (Nagasaki, Nagasaki); Dr H Eguchi, Kurume University (Kurume, Fukuoka); Dr G Urano, University of Occupational and Environmental Health (Kitakyushu, Fukuoka); and Dr J Okamura, Kyushu Cancer Center (Fukuoka, Fukuoka) for providing samples, pathology slides and clinical data.

### References

BEVILACQUA G, SOBEL ME, LIOTTA LA AND STEEG PS. (1989). Association of low *nm23* RNA levels in human primary infiltration ductal breast carcinomas with lymph-node involvement and other histopathological indicators of high metastatic potential. *Cancer Res.*, **49,** 5185–5190.

CARON H. (1995). Allelic loss of chromosome 1 and additional chromosome 17 material are both unfavourable prognostic markers in neuroblastoma. *Med. Pediatr. Oncol.*, **24,** 215–221.

CHANG, CL, ZHU X, THORAVAL DH, UNGAR D, RAWWAS J, HORA N, STRAHLER JR AND HANASH SM. (1994). *nm23-H1* mutation in neuroblastoma. *Nature*, **370,** 335–336.

EVANS AE, D'ANGIO DJ AND RANDOLPH J. (1971). A proposed staging for children with neuroblastoma. *Cancer*, **27,** 374–378.

FEINBERG AP AND VOGELSTEIN B. (1983). A technique for radiolabeling DNA restriction endonuclease fragments to high specific activity. *Anal. Biochem.*, **132,** 6–13.

FONG CT, DRACOPOLI NC, WHITE PS, MERRILL PGT, GRIFFITH RC, HOUSMAN DE AND BRODEUR GM. (1989). Loss of heterozygosity for the short arm of chromosome 1 in human neuroblastomas: correlation with N-myc amplification. *Proc. Natl Acad. Sci. USA*, **86,** 3753–3757.

FONG CT, WHITE PS, PETERSON K, SAPIENZA C, CAVENEE WK, KERM SE, VOGELSTEIN B, CANTOR AB, LOOK AT AND BRODEUR GM. (1992). Loss of heterozygosity for chromosomes 1 or 14 defines subsets of advanced neuroblastomas. *Cancer Res.*, **52,** 1780–1785.

GEHAN E. (1965). A generalized Wilcoxon test for comparing arbitrarily singly-censored samples. *Biometrika*, **52,** 203–224.

GILLES AM, PRESECAN E, VONICA A AND LASCU I. (1991). Nucleoside diphosphate kinase from human erythrocytes. *J. Biol. Chem.*, **266,** 8784–8789.

HAILAT N, KEIM DR, MELHEM RF, ZHU X, ECKERSKORN C, BRODEUR GM, REYNOLDS CP, SEEGER RC, LOTTSPEICH F, STRAHLER JR AND HANASH SM. (1991). High levels of p19/nm23 protein in neuroblastoma are associated with advanced stage disease and N-*myc* gene amplification. *J. Clin. Invest.*, **88,** 341–345.

HUMAN GENE MAPPING 11. (1991). *Cytogenet. Cell Genet.*, **58,** 1440–1569.

ISCN 1991. (1992). *Guidelines for Cancer Cytogenetics: Supplement to an International System for Human Cytogenetic Nomenclature.* Karger: Basle.

KAPLAN EL AND MEIER P. (1958). Nonparametric estimation for incomplete observations. *J. Am. Stat. Assoc.*, **53,** 457-481.

LEONE A, SEEGER RC, HONG CM, HU YY, ARBOLEDA MJ, BRODEUR GM, STRAM D, SLAMON DJ AND STEEG PS. (1993). Evidence for *nm23* RNA overexpression, DNA amplification and mutation in aggressive childhood neuroblastomas. *Oncogene*, **8,** 855-865.

MURAKAMI Y, HAYASHI K AND SEKIYA T. (1991). Detection of aberrations of the p53 alleles and the gene transcript in human tumor cell lines by single-stranded conformation polymorphism analysis. *Cancer Res.*, **51,** 3356-3361.

NAKAMURA Y, LATHROP M, O'CONNELL P, LEPPERT M, KAMBOH MI, LALOUEL JM AND WHITE R. (1989). Frequent recombination is observed in the distal end of the long arm of chromosome 14. *Genomics*, **4,** 76-81.

OKADA K, URANO T, GOI T, BABA H, YAMAGUCHI A, FURUKAWA K AND SHIKU H. (1994). Isolation of human *nm23* genomes and analysis of loss of heterozygosity in primary colorectal carcinomas using a specific genomic probe. *Cancer Res.*, **54,** 3979-3982.

PETO R AND PETO J. (1972). Asymptotically efficient rank invariant test procedures. *J. R. Stat. Soc.*, (a), **135,** 185-206.

SAWADA T, HIRAYAMA M AND NAKATA T. (1984). Mass screening for neuroblastoma in infants in Japan. *Lancet*, **2,** 271-273.

SCHLEIERMACHER G, PETER M, MICHON J, HUGOT J, VIELH P, ZUCKER J, MAGDELENAT H, THOMAS G AND DELATTRE O. (1994). Two distinct deleted regions on the short arm of chromosome 1 in neuroblastoma. *Genes Chrom. Cancer*, **10,** 275-281.

SRIVATSAN ES, YING KL AND SEEGER RC. (1993). Deletion of chromosome 11 and of 14q sequences in neuroblastoma. *Genes Chroms. Cancer*, **7,** 32-37.

STEEG PS, BEVILACQUA G, KOPPER L, THORGEIRSSON UP, TALMADGE JE, LIOTTA LA AND SOBEL ME. (1988). Evidence for a novel gene associated with low tumor-metastatic potential. *J. Natl Cancer Inst.*, **80,** 200-204.

SUZUKI T, YOKOTA J, MUGISHIMA H, OKABE I, OOKUNI M, SUGIMURA T AND TERADA M. (1989). Frequent loss of heterozygosity on chromosome 14q in neuroblastoma. *Cancer Res.*, **49,** 1094-1098.

TAKAYAMA H, SUZUKI T, MUGISHIMA T, FUJISAWA T, OOKUNI M, SCHWAB M, GEHRING M, NAKAMURA Y, SUGIMURA T, TERADA M AND YOKOTA J. (1992). Deletion mapping of chromosome 14q and 1p in human neuroblastoma. *Oncogene*, **7,** 1185-1189.

TAKEDA O, HOMMA C, MASEKI N, SAKURAI M, KANDA N, SCHWAB M, NAKAMURA Y AND KANEKO Y. (1994). There may be two tumor suppressor genes on chromosome 1p closely associated with biologically distinct subtypes of neuroblastoma. *Genes Chrom. Cancer*, **10,** 30-39.

URANO T, FURUKAWA K AND SHIKU H. (1993). Expression of nm23/NDP kinase proteins on the cell surface. *Oncogene*, **8,** 1371-1376.

VARESCO L, CALIGO MA, SIMI P, BLACK DM, NARDINI V, CASARINO L, ROCCHI M, FERRARA G, SOLOMON E AND BEVILACQUA. (1992). The *nm23* gene maps to human chromosome band 17q22 and shows a restriction fragment length polymorphism with *Bgl*II. *Genes Chrom. Cancer*, **4,** 84-88.

WEITH A, MARTINSSON T, CZIEPLUCH C, BRUDERLEIN S, AMLER LC, BERTHOLD F AND SCHWAB M. (1989). Neuroblastoma consensus deletion maps to 1p36.1-2. *Genes Chrom. Cancer*, **1,** 159-166.